# Safety and feasibility of conversion of Roux-en-Y gastric bypass to BPD/DS versus SADI: an analysis of MBSAQIP database

**DOI:** 10.1007/s00464-025-12556-w

**Published:** 2026-01-22

**Authors:** J. L. Serra, A. Estrada, Y. Rivero-Moreno, N. Hindosh, J. Choi, E. Moran-Atkin, D. Camacho

**Affiliations:** Department of Surgery, Montefiore Medical Center, New York, NY USA

**Keywords:** Bariatric surgery, Roux-en-Y gastric bypass, Biliopancreatic diversion with duodenal switch, Single anastomosis duodeno-ileal bypass, MBSAQIP

## Abstract

**Background:**

Recurrent weight gain (RWG) after Roux-en-Y gastric bypass (RYGB) is an increasingly common challenge. Biliopancreatic diversion with duodenal switch (BPD/DS) and single anastomosis duodeno-ileal bypass with sleeve gastrectomy (SADI-S) are surgical options available for achieving optimal clinical response in these patients.

**Objectives:**

This study aimed to compare the characteristics and outcomes of patients who underwent either BPD/DS or SADI-S following RYGB.

**Methods:**

An analysis of the Metabolic and Bariatric Surgery Accreditation and Quality Improvement Program (MBSAQIP) database was conducted, focusing on patients from 2020 to 2022 who underwent BPD/DS or SADI-S following a primary RYGB. Postoperative bariatric outcomes, complication rates, and weight loss metrics—including percentage of excess weight loss (%EWL), total weight loss (%TWL), and excess BMI loss (%EBMIL) at 30 days—were evaluated. A stratified analysis was also performed to assess differences between the procedures.

**Results:**

A total of 616 patients were included. 75.5% (*n* = 465) underwent BPD/DS and 24.5% (*n* = 151) SADI after RYGB. The majority of patients were female (90.7%, *n* = 559), with a mean age of 48.2 ± 9.1 years. The mean preoperative body mass index (BMI) was 47.5 ± 8.3 for patients undergoing BPD/DS and 44.5 ± 6.8 for those undergoing SADI (*p* < 0.001). Patients who underwent BPD/DS had a higher prevalence of hypertension (47% vs. 33.7%, *p* = 0.004) and ASA IV classification (12.2% vs. 1.3%, *p* < 0.001), but a lower rate of drain placement compared to those who underwent SADI (31.8% vs. 45%, *p* = 0.003). There was no statistically significant difference in terms of length of stay, operative time, or intraoperative/postoperative complications in the first 30 days. Weight loss outcomes at 30 days were comparable between the BPD/DS and SADI approaches.

**Conclusions:**

Among patients undergoing conversion after RYGB, BPD/DS and SADI demonstrated comparable short-term bariatric outcomes, with similar 30-day postoperative complication rates.

Roux-en-Y gastric bypass (RYGB) is among the most commonly performed bariatric procedures worldwide and has demonstrated durable weight loss and remission of obesity-related comorbidities. In 2022, Roux-en-Y gastric bypass (RYGB) accounted for 22.2% of the total bariatric surgical volume in the United States [1]. A recent systematic review found that most RYGB studies reported recurrent weight gain (RWG) rates within 15% of those observed in the reference sample [2], which may lead to recurrence of obesity-associated conditions such as type 2 diabetes, hypertension, and obstructive sleep apnea, and reduced quality of life.

Revisional bariatric surgery has therefore emerged as an important consideration in this population. Revisional procedures have increased over the past decade, reflecting obesity’s chronic nature and inconsistent treatment outcomes [1]. Several options exist for revision of RYGB after failure including endoluminal approach [3], revision of the pouch [4], conversion to a distal RYGB [5], and conversion to a biliopancreatic diversion with duodenal switch (BPD/DS) or single anastomosis duodeno-ileal bypass with sleeve gastrectomy (SADI-S) [6, 7]. Among the available revisional options, BPD/DS and SADI-S have gained increasing attention for their potent metabolic benefits and durable weight loss effects. BPD/DS is particularly recognized for its strong impact on long-term weight reduction and remission of obesity-related conditions such as type 2 diabetes, especially in patients with BMI ≥ 50 kg/m^2^ [8] Similarly, SADI-S has emerged as an effective alternative that combines significant weight loss with improvements in metabolic parameters, including better glycemic and lipid control [9, 10]. Despite the growing use of both techniques, there is limited evidence directly comparing the safety and short-term outcomes of BPD/DS versus SADI-S following RYGB.

Most data in the literature compare both techniques as a revisional procedure after sleeve gastrectomy (SG), but not after RYGB [11]. Understanding these outcomes is critical to guiding surgical decision-making, patient counseling, and assessing the perioperative risks.

Therefore, this study aims to analyze and compare the perioperative characteristics, complication rates, and short-term weight loss outcomes of patients undergoing conversion from RYGB to either BPD/DS or SADI-S.

## Materials and methods

This was a retrospective cohort study analyzing the Metabolic and Bariatric Surgery Accreditation and Quality Improvement Program (MBSAQIP) database, focusing on patients from January 1, 2020 to December 31, 2022, who underwent BPD/DS or SADI-S following a primary RYGB. The MBSAQIP database is a national clinical registry that captures detailed data from accredited bariatric surgery centers across North America. The dataset includes preoperative, intraoperative, and 30-day postoperative outcomes for patients undergoing metabolic and bariatric surgery.

Demographic data such as age, gender, type of procedure, preoperative body mass index (BMI), preoperative comorbidities such as diabetes mellitus (DM), hypertension (HTN), hyperlipidemia (HLD), obstructive sleep apnea (OSA), gastroesophageal reflux disease (GERD), smoking status, and American Society of Anesthesiologists (ASA) classification were extracted from the database.

Our primary outcomes included 30-day postoperative complications. The following variables we included: surgical site infection (SSI), anastomotic leak, wound dehiscence, pneumonia, pulmonary embolism (PE), acute kidney injury (AKI), myocardial infarction (MI), sepsis, gastrointestinal bleeding, death, reoperations, readmissions, and emergency department visits within 30 days.

The secondary outcomes of the study included: operative variables such as surgical approach (open, laparoscopic, robotic), operative time, conversion to open, and drain placement. Additionally, weight loss outcomes expressed as percent excess weight loss (%EWL), total weight loss (%TWL), and excess BMI loss (%EBMIL) at 30 days postoperatively were evaluated.

Patients were excluded if they had missing data on key outcome variables, incomplete demographic or operative data, or revisional procedures other than BPD/DS or SADI-S.

Quantitative variables were expressed as median and standard deviation (SD) if normally distributed, and qualitative variables as proportions. The Chi-square was used for categorical variables, and the t-student test was used for quantitative variables. The statistical software IBM SPSS Statistics for Windows, Version 29.0.2.0 (Armonk, NY: IBM Corp) was used to conduct statistical tests to assess for statistical significance (*p* < 0.05).

This study was reviewed and deemed exempt by the Institutional Review Board (IRB) due to the anonymity of the data and was conducted following the 1964 Helsinki Declaration and its later amendments or comparable ethical standards. This study was conducted following the guidelines and recommendations of the STROBE guidelines.

## Results

A total of 616 patients who underwent either BPD/DS or SADI-S following a primary RYGB between 2020 and 2022 were included in the analysis. Of these, 465 patients (75.5%) underwent conversion to BPD/DS, while 151 patients (24.5%) underwent conversion to SADI-S. The majority of patients were female (90.7%), with a mean age of 48.2 ± 9.1 years. The mean preoperative BMI was 47.5 ± 8.3 for patients undergoing BPD/DS and 44.5 ± 6.8 for those undergoing SADI (*p* < 0.001). The prevalence of hypertension (47% vs. 33.7%, *p* = 0.004) and ASA class IV status (12.2% vs. 1.3%, *p* < 0.001) was also higher in the BPD/DS group. There were no statistically significant differences in age, diabetes mellitus, hyperlipidemia, GERD, smoking status, or most other comorbidities.

There were no significant differences between the two groups in terms of operative time (211.6 ± 82.9 min vs. 211.9 ± 86.9 min, *p* = 0.429) or hospital length of stay (3.0 ± 4.6 vs. 3.1 ± 4.1 days, *p* = 0.747). Patients in the SADI-S group had higher rates of drain placement (45% vs. 31.8%, *p* = 0.003). The majority of surgeries in both groups were performed laparoscopically or robotically, with no statistically significant differences in surgical approach or conversion rates.

The overall 30-day complication rate was similar between the two groups (17.2% in BPD/DS vs. 18.5% in SADI-S, *p* = 0.706). Anastomotic leaks were reported in 5.8% of BPD/DS and 5.9% of SADI-S patients (*p* = 0.944). Gastrointestinal bleeding occurred in 3.0% of BPD/DS versus 1.3% of SADI-S patients (*p* = 0.388), while intraoperative or postoperative transfusion requirements were 3.8% and 2.6%, respectively (*p* = 0.482). Rates of other complications—including surgical site infections, anastomotic leak, pneumonia, pulmonary embolism, acute kidney injury, and sepsis—were also not significantly different. There were 4 reported deaths (0.6%), all occurring in the BPD/DS group (*p* = 0.252).

Rates of reoperation (7.9% vs. 7.2%, *p* = 0.788), readmission (14.8% vs. 13.9%, *p* = 0.778), and emergency department visits (12% vs. 11.2%, *p* = 0.795) within 30 days were comparable between the two groups.

Weight loss outcomes at 30 days were also comparable between the BPD/DS and SADI-S approaches. The mean percentage of excess weight loss (%EWL) was 11.4 ± 9.7% in the BPD/DS group versus 14.4 ± 7.8% in the SADI-S group (*p* = 0.462). Total weight loss (%TWL) was 6.0 ± 5.1% for BPD/DS and 7.1 ± 3.6% for SADI-S (*p* = 0.323). Similarly, percentage excess BMI loss (%EBMIL) was 12.9 ± 11.7% in BPD/DS patients compared to 16.3 ± 9.5% in those who underwent SADI-S (*p* = 0.531). None of the differences reached statistical significance, indicating that both procedures achieved similar early weight loss effectiveness (Table 1).

**Table 1:** Characteristics of bariatric patients who underwent BPD/DS vs. SADI after RYGB

Characteristics ^a^ ±	Total (*n = *616)	BPD/DS (*n* = 465)	SADI (*n* = 151)	*p* value
Sex—female	559 (90.7)	417 (89.6)	142 (94)	**0.039**
Age	48.2 ± 9.1	48.5 ± 9	47.8 ± 9	0.867
BMI before surgery	46.8 ± 8	47.5 ± 8.3	44.5 ± 6.8	** < 0.001**
DM	94 (15.2)	71 (15.2)	23 (15.2)	0.991
Chronic use of steroids/immunosuppression	26 (4.2)	23 (4.9)	3 (1.9)	0.116
HTN	270 (43.8)	219 (47)	51 (33.7)	**0.004**
HLD	86 (13.9)	68 (14.6)	18 (11.9)	0.405
Current smoker within 1 year	34 (5.5)	25 (5.3)	9 (5.9)	0.784
COPD	10 (1.6)	5 (1)	5 (3.3)	0.058
History of PE	19 (3)	17 (3.6)	2 (1.3)	0.149
History of DVT	20 (3.2)	17 (3.6)	3 (1.9)	0.314
Therapeutic anticoagulation	26 (4.2)	21 (4.5)	5 (3.3)	0.522
OSA	189 (30.6)	162 (34.8)	27 (17.8)	8.642
GERD	169 (27.4)	127 (27.3)	42 (27.8)	0.904
ASA II	64 (10.3)	41 (8.8)	23 (15.2)	**0.024**
ASA III	493 (80)	367 (78.9)	126 (83.4)	0.228
ASA IV	59 (9.5)	57 (12.2)	2 (1.3)	** < 0.001**
Open	25 (4)	23 (4.9)	2 (1.3)	0.050
Laparoscopic	350 (56.8)	257 (55.2)	93 (61.5)	0.108
Robotic approach	242 (39.2)	186 (40)	56 (37)	0.524
Conversion to open	5 (0.8)	3 (0.6)	2 (1.3)	0.418
Drain placement	216 (35)	148 (31.8)	68 (45)	**0.003**
Length of the procedure (min)	211.6 ± 82.9	211.6 ± 81.7	211.9 ± 86.9	0.429
Hospital Stay (days)	3.1 ± 4.5	3 ± 4.6	3.1 ± 4.1	0.747
Intraop/Postop complications in the first 30 days	108 (17.5)	80 (17.2)	28 (18.5)	0.706
SSI	55 (8.9)	38 (8.1)	17 (11.2)	0.247
Anastomotic leak	26 (4.2)	27 (5.8)	9 (5.9)	0.944
Wound dehiscence	2 (0.3)	2 (0.4)	0 (0)	0.419
Post pneumonia	11 (1.7)	8 (1.7)	3 (1.9)	0.830
PE	4 (0.4)	3 (0.4)	1 (0.6)	0.798
AKI	8 (1.2)	7 (1.5)	1 (0.6)	0.426
MI	1 (0.1)	0 (0)	1 (0.6)	0.079
Post-Op sepsis	19 (3)	16 (3.4)	3 (1.9)	0.369
Intraoperative or postoperative transfusion	22 (3.5)	18 (3.8)	4 (2.6)	0.482
GI tract bleed	16 (2.6)	14 (3)	2 (1.3)	0.388
Reoperation 30 days	48 (7.7)	37 (7.9)	11 (7.2)	0.788
Readmission 30 days	90 (14.6)	69 (14.8)	21 (13.9)	0.778
Emergency visit	73 (11.8)	56 (12)	17 (11.2)	0.795
%EWL at 30 days	11.9 ± 9.4	11.4 ± 9.7	14.4 ± 7.8	0.462
%TWL at 30 days	6.2 ± 4.9	6 ± 5.1	7.1 ± 3.6	0.323
% EBMIL at 30 days	13.5 ± 11.4	12.9 ± 11.7	16.3 ± 9.5	0.531
Death	4 (0.6)	4 (0.8)	0 (0)	0.252

*BMI* body mass index, *OSA* Obstructive sleep apnea, *ASA* American Society of Anesthesiologists, *PE* Pulmonary Embolism, *DVT* deep venous thromboembolism, *AC* anticoagulation, *DM* diabetes mellitus, *HTN* hypertension, *HLD* hyperlipidemia, *GERD* gastroesophageal reflux disease, *COPD* chronic obstructive pulmonary disease, %*EWL* percentage of excess weight loss, %*TWL* percentage of total weight loss, %*EBMIL* Percentage excess BMI loss

^a^Continuous data are shown as the mean ± standard deviation and categorical data as number (%)

^*^Chi-square

^†^T-Student

Bold values are statistically significant

## Discussion

The gathered data compares the short-term outcomes of patients undergoing conversion from Roux-en-Y gastric bypass (RYGB) to either biliopancreatic diversion with duodenal switch (BPD/DS) or single anastomosis duodeno-ileal bypass with sleeve gastrectomy (SADI-S). Our findings demonstrate that both procedures are feasible revisional options following failed RYGB, with comparable 30-day complication rates and short-term weight loss outcomes.

The complication rate of converting RYGB to BPD/DS has been examined primarily in small cohorts and single-center studies. Roulet et al. investigated the complication rate of RYGB-to-BPD/DS conversion, reporting a 30-day complication rate of 25.7%, with 14.8% of patients requiring reoperation [12]. A similar single-center study of RYGB to SADI conversion reported a 30-day complication rate of 50% (*n* = 6), including one leak and one hemoperitoneum [13]. Our cohort showed a lower overall 30-day complication rate (17.2%) between the two procedures, likely reflecting more recent data and the tendency toward fewer complications as new surgical techniques are adopted.

In terms of anastomotic leaks for BPD/DS, current data reports that the incidence of a gastric or duodenal leak following primary BPD/DS is 1.14% [14]. Interestingly, for patients undergoing conversion of RYGB to BPD/DS, one study with a small cohort of 17 patients reported a leak rate of 11.7% (*n* = 2) [15]. The present study reported a lower leak rate of 5.8%, likely related to a much higher number of participants. On the other hand, the anastomotic leak rate for primary SADI-S is reported as 2.2% in a recent MBSAQIP analysis from 2020 [16]. In a recent single-center study by Sanchez-Pernaute et al. five patients underwent conversion from RYGB to SADI-S using a staged approach, with no anastomotic leaks reported [17]. Our cohort found an increased rate of anastomotic leaks for conversion of RYGB to SADI (5.9%). This may be attributed to a more conservative two-stage approach, particularly given that this is the same group of experts from Spain who first described the SADI technique in 2007.

Notably, the anastomotic leak rates in both groups (5.8% and 5.9%) are higher than those reported in primary BPD/DS and SADI-S procedures [14, 16]. This difference likely reflects the increased technical demands of revisional surgery, altered anatomy from RYGB, presence of adhesions, and higher-risk patient characteristics including elevated BMI and ASA classification. While these rates remain within expected ranges for complex revisional operations, they highlight the need for meticulous technique and heightened awareness of leak risk in this setting.

Regarding overall readmission within 30 days between the two procedures, Surve et al. [18] also reported that 15.6% of their patients required readmission following the conversion. In our study, we reported a similar overall readmission rate (14.6%), with minimal difference between the two techniques. Similarly, Moon et al. reported that 13.3% (*n* = 2) of their patients underwent a reoperation within 30 days [6]. This may suggest that neither the number of stages nor the type of procedure was associated with the complication rate. We reported a lower overall reoperation rate at 30 days (7.7%), with minimal difference observed between the two groups.

With respect to complications, it is important to note that one of the most concerning adverse outcomes after BPD/DS is the development of malnutrition and hepatic impairment, which typically develops several months postoperatively and cannot be evaluated in this study. Current literature reports rates as high as 10% [19].

Prior small, single-center studies have compared both techniques and found no statistically significant difference in mean %EWL at 12 and 24 months between them [18]. In our study, in terms of early weight loss, SADI-S showed a numerically higher—but not statistically significant—effect across all metrics (%EWL, %TWL, %EBMIL). These findings are consistent with prior reports [6] suggesting comparable short-term weight loss efficacy between the two procedures, with SADI-S offering potential technical advantages in the revisional setting. However, the lack of a significant difference reinforces that both BPD/DS and SADI-S are similarly effective in promoting early postoperative weight reduction after RWG following RYGB. In a smaller cohort, Moon et al. [6] showed that conversions to duodenal switch resulted in an EBMIL of 57.7% at 24 months, suggesting that the weight loss from these revisional procedures can be sustained up to 2 years. Nevertheless, studies assessing long-term outcomes are still warranted.

The major strength of this study is its use of a large, multicenter dataset (MBSAQIP), which enhances generalizability across diverse surgical practices. The inclusion of over 600 patients undergoing revisional procedures represents one of the largest cohorts to date.

This study has several limitations. Its retrospective design inherently limits the ability to establish causality and may be subject to selection bias. Additionally, the MBSAQIP database reports only 30-day postoperative outcomes, which restricts the assessment of medium- and long-term efficacy, including nutritional complications and the durability of weight loss. Furthermore, detailed information on operative techniques—such as limb lengths and anastomotic methods—as well as surgeon experience was not available.

## Conclusion

In conclusion, this national cohort analysis demonstrates that both BPD/DS and SADI-S show comparable short-term outcomes when performed as revisional procedures following RYGB. Although overall perioperative complications did not differ between groups, the approximately 6% anastomotic leak rate observed in both cohorts highlights the greater complexity and risk inherent to revisional bariatric surgery relative to primary operations. Careful patient selection, meticulous operative technique, and heightened postoperative vigilance are therefore essential when considering these procedures. Further studies with long-term follow-up are needed to evaluate sustained weight loss, nutritional consequences, and quality of life outcomes.
